# Genome-wide DNA methylation changes associated with olfactory learning and memory in *Apis mellifera*

**DOI:** 10.1038/s41598-017-17046-1

**Published:** 2017-12-05

**Authors:** You Li, Li-Zhen Zhang, Yao Yi, Wan-Wan Hu, Ya-Hui Guo, Zhi-Jiang Zeng, Zachary-Y. Huang, Zi-Long Wang

**Affiliations:** 1 Honeybee research institute, Jiangxi Agricultural University, Nanchang, Jiangxi 330045 China; 2 Department of Entomology, Michigan State University, East Lansing, Michigan 48824 USA

## Abstract

The honeybee is a model organism for studying learning and memory formation and its underlying molecular mechanisms. While DNA methylation is well studied in caste differentiation, its role in learning and memory is not clear in honeybees. Here, we analyzed genome-wide DNA methylation changes during olfactory learning and memory process in *A. mellifera* using whole genome bisulfite sequencing (WGBS) method. A total of 853 significantly differentially methylated regions (DMRs) and 963 differentially methylated genes (DMGs) were identified. We discovered that 440 DMRs of 648 genes were hypermethylated and 274 DMRs of 336 genes were hypomethylated in trained group compared to untrained group. Of these DMGs, many are critical genes involved in learning and memory, such as *Creb*, *GABA*_*B*_ *R* and *Ip3k*, indicating extensive involvement of DNA methylation in honeybee olfactory learning and memory process. Furthermore, key enzymes for histone methylation, RNA editing and miRNA processing also showed methylation changes during this process, implying that DNA methylation can affect learning and memory of honeybees by regulating other epigenetic modification processes.

## Introduction

Honeybees (*Apis mellifera*) are social insects with important economic and ecological value due to their pollination services. They are able to distinguish different colors^1^, odors^2^, even the relationship between objects, such as, sequential order^3^, and the upper and lower^4^. Even more astonishing is their ability to learn abstract rules and concepts^5,6^. Due to their remarkable abilities in learning and memory and the relative simple structure of their brains, honeybees are considered a good model for neurobiology. But the molecular mechanisms underlying the learning and memory process of honeybees are not well understood.

Epigenetic modifications, such as DNA methylation^7,8^, histone modifications^9,10^, and miRNA processing^11,12^, have been shown to be involved in learning and memory processes in vertebrates. DNA methylation plays a critical role in long-term memory formation in many organisms and different learning paradigms^8^. The change of DNA methylation levels, which is regulated mainly by the activity of DNA methyltransferase (DNMTs), directly regulates the expression level of genes involved in memory formation in the brain^13,14^.

The honeybee genome possesses a complete, functional DNA methylation system, which includes an ortholog of *Dnmt3* and two orthologs of *Dnmt1* (*AmDnmt1a* and *AmDnmt1b*)^15^. Moreover, all these three DNMTs have catalytic activity. DNA methylation is regarded as the main mechanism regulating queen-worker caste differentiation in honeybees, and many studies have surveyed DNA methylation differences between these two castes^16–20^. DNA methylation is also reported to be closely associated with learning and memory processes in honeybees^21–23^. Inhibiting the activity of DNMT in honeybees using zebularine revealed that DNA methylation mediates long-term memory formation in honeybees after associative learning, but not short-term memory formation^21,22^. By measuring the methylation of 30 memory associated genes in honeybees, Biergans *et al*.^23^ found that, during memory formation process, memory associated genes are regulated by a temporally complex epigenetic mechanism.

Even though it is known DNA methylation is involved in long-term memory formation in *A. mellifera*, the actual pattern of DNA methylation after learning and memory has not been studied in honeybees. In this process, how many genes are methylated? To what extent does DNA methylation affect gene expression? Here, we analyzed the genome-wide DNA methylation changes following olfactory learning and memory in *A. mellifera* through WGBS method, and identified many DMRs and associated genes. Our results suggest extensive involvement of DNA methylation in honeybee olfactory learning and memory.

## Results

### DNA methylation patterns

After sequencing, about 72,434,814 and 76,497,724 raw reads with a Q20 value of more than 92% were generated for the two samples, respectively (Table 1). After removing low-quality reads, 58,669,954 (7.34 Gb) and 64,500,902 (8.06 Gb) clean reads which were more than 25 × coverage of the 285 Mb* A. mellifera* reference genome remained, and were mapped to the genome. Finally, 56.95% and 61.10% of the reads were uniquely mapped to the honeybee genome and more than 77% of the total clean reads have a coverage of >5 × on the genome (Table 1). The BS conversion rates are more than 99.83% in each sample, indicating high T-C conversion during bisulfite treatment.

**Table 1:** Data generated by whole genome bisulfite sequencing.

	Trained	Untrained
Raw reads	72434814	76497724
Raw bases (G)	9.04	9.56
Clean reads	58669954	64500902
Clean bases (G)	7.34	8.06
Q20 (%)	92.91%	93.16%
BS Conversion Rate (%)	99.85%	99.83%
Total mapped reads	33414826	39408700
Mapping rate (%)	56.95%	61.10%
Duplication rate (%)	3.4%	3.4%
5× coverage (%)	78.50%	77.30%

**Figure 1:**
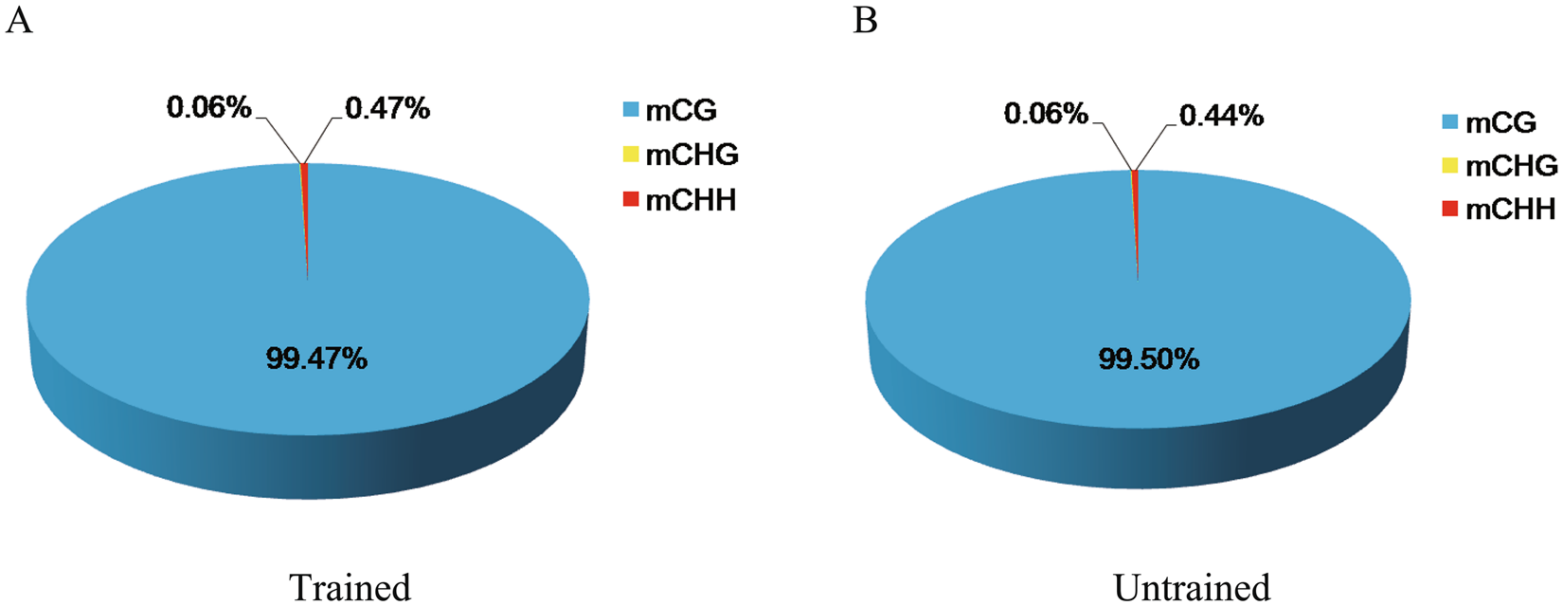
The distribution (%) of mCs in CG, CHG and CHH contexts.

A total of 139,430 and 140,997 methylated cytosines (mCs) were detected in trained and untrained groups, both occupying about 0.19% of all the cytosine sites in the honeybee genome (Table 2). Of these mCs, the percentages of mCs in CG, CHG (H represents A, C or T) and CHH contexts in the trained group were 99.47%, 0.06%, 0.47% (Fig. 1A), occupying 0.6829%, 0.0010%, 0.0014% of the genome-wide CG, CHG and CHH sites respectively (Table 2); the corresponding numbers in the untrained group were 99.50%, 0.06%, 0.44% (Fig. 1B), occupying 0.6908%, 0.0010%, 0.0014% of the genome-wide CG, CHG and CHH sites respectively (Table 2).

**Table 2:** The genome-wide percentages of methylated CG, CHG and CHH in the trained and untrained groups.

	Trained	Untrained
mC percent (%)	0.1861%	0.1882%
mCG percent (%)	0.6829%	0.6908%
mCHG percent (%)	0.0010%	0.0010%
mCHH percent (%)	0.0014%	0.0014%

### Sequence preferences flanking the methylated C sites

**Figure 2:**
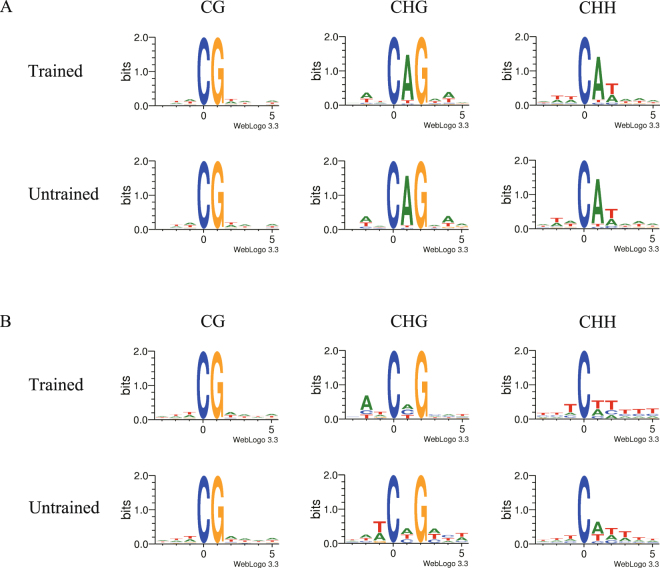
Sequence preferences around the methylated C sites in CG, CHG, and CHH contexts. (**A**) High methylation regions. (**B**) Low methylation regions.

We analyzed the relationship between sequence context and methylation preference. The same criteria used by Lister *et al*.^24^ were adopted to define the methylation level. That is, for CG context, C sites with methylation level exceeding 75% were defined as high methylation sites, and C sites with methylation level lower than 75% were defined as low methylation sites; while for non-CG context, C sites with methylation level exceeding or under 25% are defined as high or low methylation sites, respectively. The sequence characteristics of 9-mer sequences around the methylated C sites under different context sequence were analyzed (Fig. 2). In the CG contexts of both high and low methylation regions, it showed no significant difference between the trained and untrained groups in the sequence enrichment based on the genomic regions, and “-TACGTA-A” and “ATTCGAAA(T)A” were the most preferred sequences around the methylated C sites in these two type of regions. While in the non-CG context the methylation showed an obvious sequence context preference, especially in the low methylation regions, in which “CATCAGCAT” and “TATCAGACT”, “TTTCTTTTT” and “TTTCATTTTT” are the most preferred sequences for the trained and untrained groups in CHG context and CHH context respectively. In the high methylation regions, the largest difference between the trained and untrained groups both in CHG and CHH context was the 3rd base of the 9-mer sequences which was closely adjacent to the methylated C site. These results suggest that methylation in non-CG sites changed greatly with a sequence preference mode during olfactory learning process in honeybees.

### DNA methylation level in different genome components

**Figure 3:**
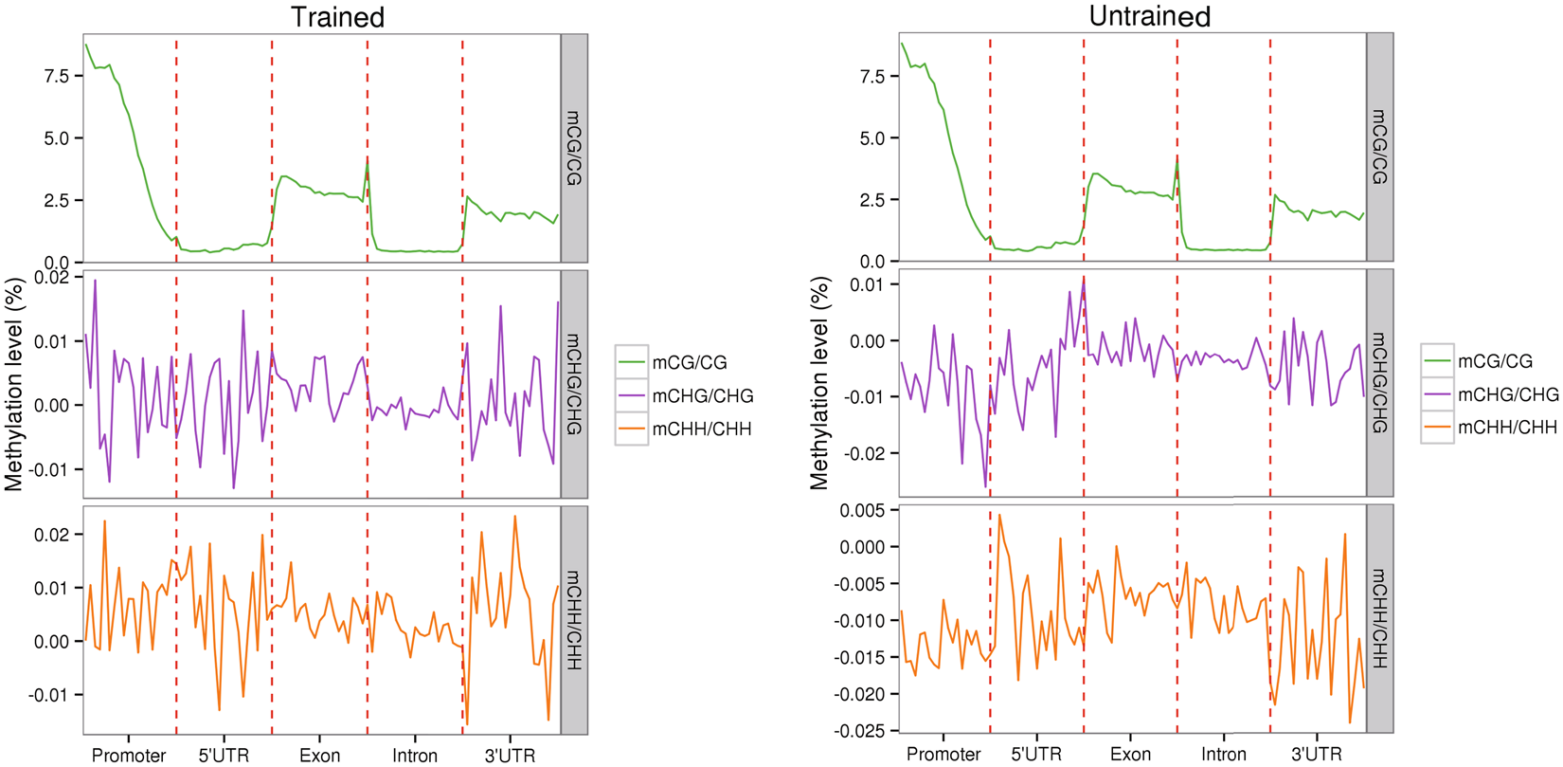
DNA methylation levels of different functional regions between the trained and untrained groups.

The DNA methylation level in general showed diverse distributions in different functional genomic regions. Thus, we investigated the DNA methylation level in five different genome components including promoter, 5′UTR, exon, intron and 3′UTR, using sliding window analysis. As shown in Fig. 3, the trained group and the untrained group showed similar pattern of methylation level distribution. In the CG context, the promoter region in which the methylation level was gradually decreased along the promoter overall showed the highest methylation level compared to other genome components, followed by the exon region, and the next was the 3′UTR; the 5′UTR and intron regions exhibited a very low level of methylation. In the CHG and CHH context, the distributions of methylation level were irregular in both trained and untrained groups.

### Differentially methylated regions and related genes

**Figure 4:**
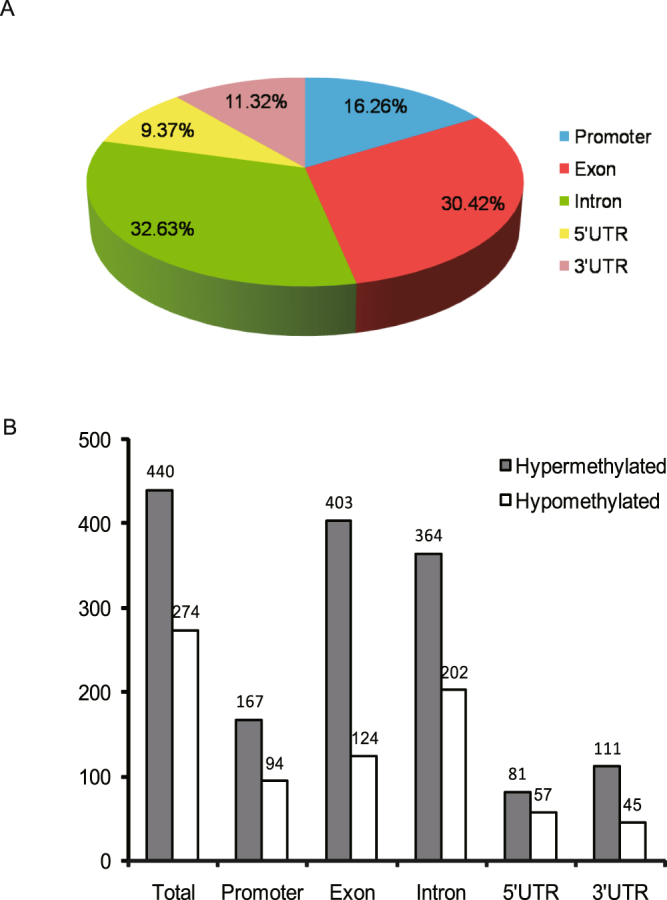
The distribution of DMRs in different genomic components. (**A**) Percentage of DMRs in different genomic components. (**B**) The number of hypermethylated and hypomethylated DMRs in different genomic components. Many DMRs were counted into more than two components because these DMRs cover multiple genomic components.

**Figure 5:**
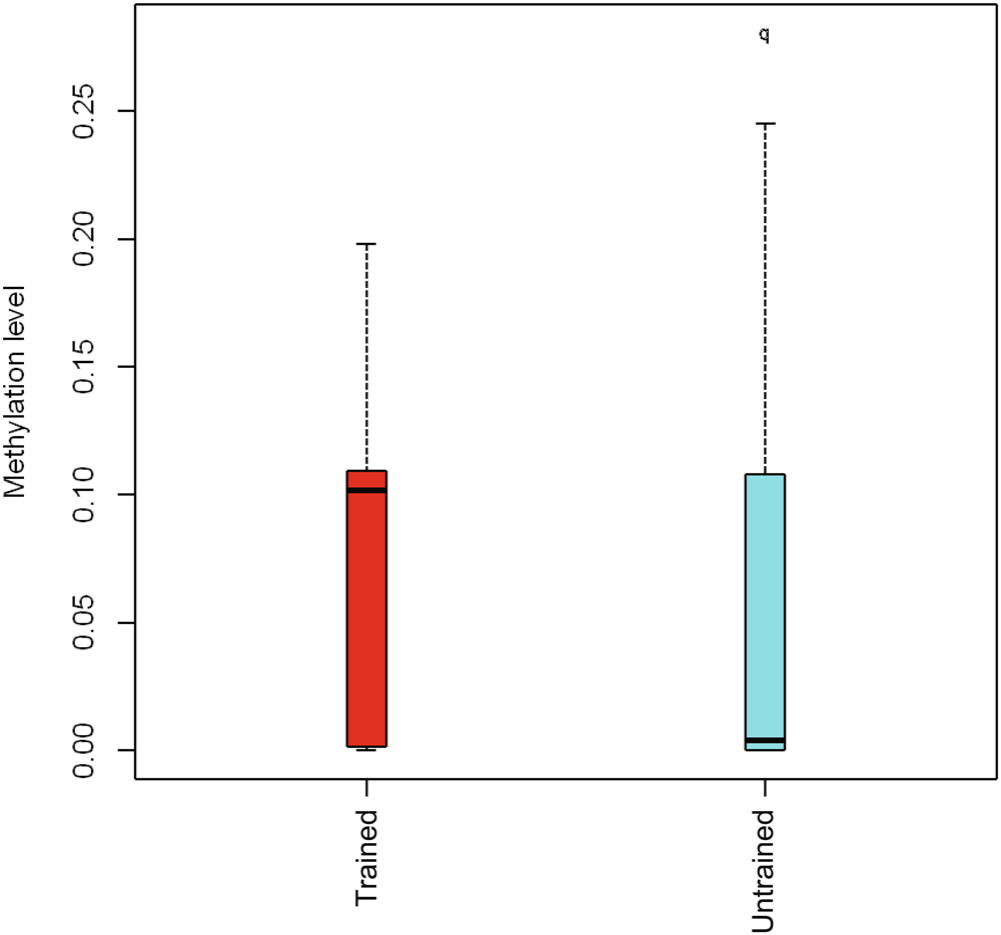
Methylation levels of DMRs in the trained and untrained groups. Boxes represent quartiles 25–75%; black lines within boxes represent median of the distribution (quartile 50%).

Differentially methylated regions between the trained and untrained groups were identified using swDMR software with rigorous parameters. A total of 853 DMRs throughout the whole genome were identified (Table S2). In order to explore the relationship between DNA methylation and gene transcription, we annotated all these DMRs using the genomic location of each DMR and the annotation information of the genome structure of *A. mellifera*. As a result, 714 DMRs belonging to 963 genes were annotated (Table S3). Of these, 440 DMRs corresponding to 648 genes were hypermethylated and 274 DMRs corresponding to 336 genes were hypomethylated in trained group compared to untrained group. These DMRs were mainly distributed in introns and exons with a percentage of 30.42% and 32.63% respectively (Fig. 4A), moreover, in all the five functional components the number of hypermethylated DMRs were larger than that of hypomethylated (Fig. 4B). Boxplot analysis of DMRs indicated that the methylation level of the trained group was higher than that of the untrained group (Fig. 5).

We paid more attention to those genes already reported to be involved in learning and memory and found many such genes from the DMG list, including cAMP response element-binding (*Creb*), metabotropic GABA-B receptor (*GABA*_*B*_ *R*), and inositol 1,4,5-triphosphate kinase (*Ip3k*), and so on.

### GO and KEGG analysis of DMGs

GO and KEGG pathway analysis were performed to analyze the significant function and pathways of the DMGs. All the DMGs were mapped to terms in the GO database and compared with the whole genome background. For all the DMGs, just the GO term “binding” with 569 DMGs in the categories of the molecular function was significantly enriched (corrected p value < 0.05), implying that a wide range of genes experienced transcriptional regulation during the process of learning and memory (Table S4). For the hypomethylated genes, 151 GO terms was significantly enriched (corrected p value < 0.05), whilst no term showed significant enrichment for the hypermethylated genes. In the KEGG analysis, no pathway was significantly enriched (corrected p value < 0.05).

### Association analysis between the DMGs and the differentially expressed genes (DEGs)

**Figure 6:**
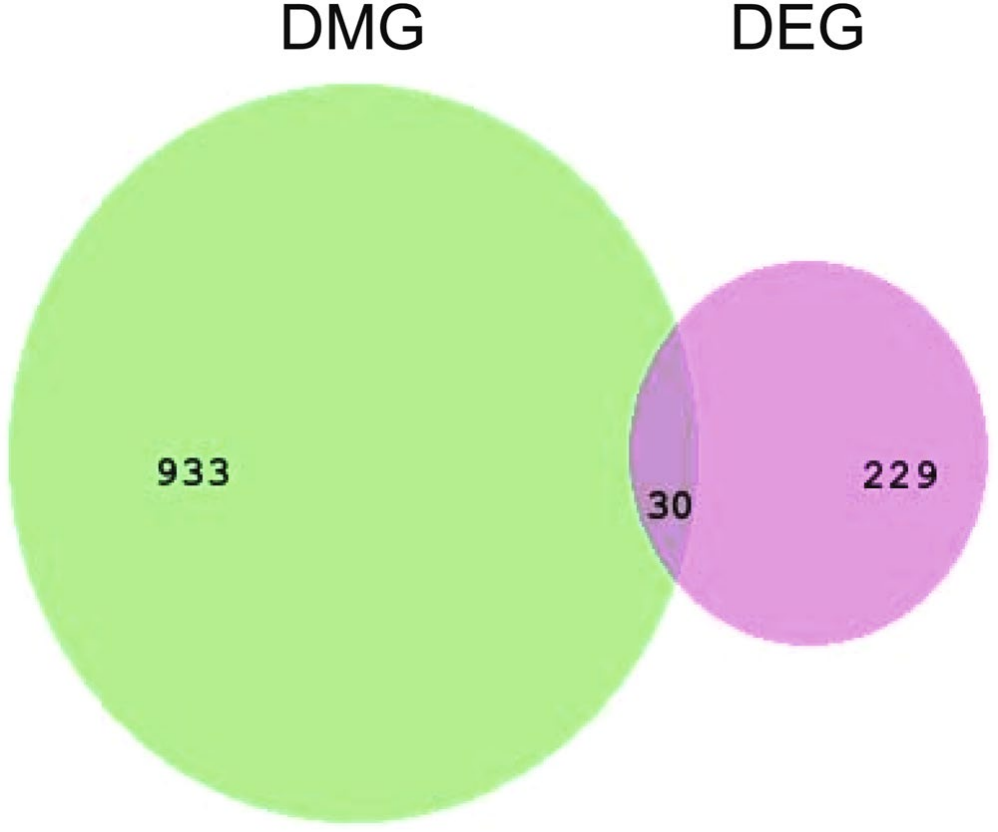
Numbers of DMGs and DEGs between the trained and untrained groups. DMG: differentially methylated gene; DEG: differentially expressed gene.

To explore the relationship between these DMGs and the DEGs found at the transcriptome level, we compared DMGs with the differentially expressed genes (DEGs) reported in our previous research^25^. In that study, we identified 259 DEGs associated with the process of honeybee olfactory learning and memory by digital gene expression analysis. As a result, we found 30 overlapped genes corresponding to 37 DMRs (Fig. 6, Table S5), including two important learning and memory related genes: *Tomosyn* and *synaptojanin 1*. Of the 37 DMRs, 20 were hypermethylated and 17 hypomethylated. Usually, hypermethylation cause down-regulation of gene transcription, we analyzed the methylation state of the DMRs within the DEGs, and found that 16 DMGs containing hypermethylated regions are down-regulated expression after olfactory learning.

## Discussion

### DNA methylation tends to represses transcription of olfactory learning and memory related genes

To analyze the effect of olfactory learning and memory on DNA methylation, we surveyed the genome-wide DNA methylation changes during this process using WGBS method. Many differentially methylated regions and associated genes were identified. Of the 714 DMRs of 963 genes, more DMRs were hypermethylated after training. Specifically, 167 of the 261 DMRs covering promoter region were hypermethylated (Fig. 4B), suggesting that transcription of most DMGs might be down-regulated during this process since DNA methylation usually represses gene transcription^26–29^, especially methylation in the promoter^26–28^ and the first exon regions^26,29^, which is in accordance with our previous observations^25,30^ as well as the results from another research group^31^ that most differentially expressed genes are down-regulated after olfactory learning and memory training. Our results suggest that DNA methylation tends to repress gene transcription after olfactory learning and memory in honeybees, although we just found 16 down-regulated hypermethylation-region containing genes in the DMGs and DEGs comparison analysis. The main reason for this relatively low number might be that the number of DEGs detected in our previous study was relatively small since there were just 259 DEGs.

### Key genes involved in learning and memory pathways

The learning and memory process contains three main signal transduction pathways, including cAMP-PKA, Ca^2+^-CaMK IV and MAPK pathways^32^. We analyzed key genes in these three signal pathways and found several such genes from the 963 DMGs, which were *Creb* (XM_623343.3), *GABA*_*B*_ *R* (XM_394454.2) and *Ip3k* (NM_001014992.1). DMRs related to all these genes were hypermethylated after training.

*Creb* is an evolutionarily highly conserved gene critical in learning and memory process in a wide range of animals and is the central collection point downstream of the cAMP-PKA, Ca^2+^-CaMK IV and MAPK signal transduction pathways^32^. In mammalian, CREB generally functions as a positive regulator in memory formation through activating the expression of its downstream genes^33,34^, and several genes, including *C-fos*, *Arc* and *Bdnf*, are reported to be its targets^35–37^. In *Drosophila*, however, *Creb* produces multiple isoforms by alternative splicing, and two of the isoforms, dCREB2-a and dCREB2-b, are functional antagonist, acting as activator and repressor respectively in long-term memory formation^38–40^. Like that in *Drosophila*, the honeybee *Creb* also has multiple alternative splicing isoforms which can be known from the mRNA sequences deposited in GenBank database. We found that the *Creb* related DMR in our study was hypermethylated after learning and memory and was located in the intron region. It is possible that this hypermethylated DMR regulates alternative splicing of *A. mellifera Creb* gene because there are evidence that DNA methylation in introns can modulate alternative splicing^41,42^, but further experiments are needed to verify this.

The metabotropic GABA-B receptor is the mediator of the inhibitory effects of the GABA which is the main inhibitory neurotransmitter in the brain^43^. By binding to GABA, GABA_B_R can mediate slow and sustained inhibitory responses through downstream Ca_2_+/K+ channels^43^. The hypermethylation of *GABA*_*B*_ *R* related DMR after learning and memory training might cause down-regulation of *GABA*_*B*_ *R* during learning and memory process. *Ip3k* is the common downstream target of *Pka*, *Pkc* and *CamkII*, which are important genes in the learning and memory signal transduction pathways^44^. PKA and CaMKII are positive regulators of *Ip3k* ^45,46^, and PKC is a negative regulator^47^. Research showed that IP3K-A plays a critical role in the spatial memory^48^.We found that *Ip3k* related DMR was hypomethylated, which might cause up-regulation of *Ip3k* expression, suggesting that IP3K might be a positive regulator during the learning and memory process.

### Other genes involved in learning and memory process

In addition to the above mentioned critical genes, from the DMGs we also found several other genes reported to be involved in learning and memory process, they were *synaptojanin 1* (XM_395173.4), *syntaxin 1 A* (XM_393760.4), syntaxin-binding protein (*Tomosyn*, XM_391820.4), synaptosomal-associated protein (*Snap-25*, XM_394588.4), glutamate-gated chloride channel (*GluCl*, NM_001077809.1), and cGMP-dependent protein kinase foraging gene (*For*, NM_001011581.1). Of them, DMR related to *synaptojanin 1* and one of the two DMRs related to *GluCl* were hypermethylated, while DMRs related to other genes were hypomethylated.

Synaptojanin 1 is a phosphoinositide phosphatase acting as a key negative regulator controlling the levels of the phosphatidylinositol-4,5-bisphosphate in the nervous tissue^49^. Down-regulating the expression of *synaptojanin 1* in mice through transgenic method reduces cognitive deficits of mice^50^. *Synaptojanin 1* related DMR was hypermethylated in this study and its expression was down-regulated after learning and memory in our previous study, implying that DNA methylation might has repressed its expression which will further promote the synthesis of the phosphatidylinositol-4,5-bisphosphate. These results are consistent with its role as a negative regulator of phosphatidylinositol-4,5-bisphosphate.

Syntaxin 1 A is an integral membrane protein playing a crucial role in neurotransmitter release by interacting with synaptobrevin (VAMP) and SNAP-25^51^. Knocking out *syntaxin 1 A* in mice leads to impairment of its consolidation and extinction of conditioned fear memory as well as the long-term potentiation in the hippocampal slice^52^, suggesting that syntaxin 1 A acts as a positive regulator in learning and memory. SNAP-25 is a 25 kDa protein involved in regulating synaptic vesicle exocytosis and voltage-gated calcium channels activity^53^. Experiments in mice indicated that SNAP-25s distributed in different region of hippocampal have different biological functions in learning and memory^54,55^. *For* encodes a cyclic guanosine-3,5-monophosphate (cGMP)-dependent protein kinase (PKG). It has been widely reported to play a role in food-search behavior of *Drosophila* ^56^. But studies in *Drosophila* showed that it is also required for visual learning^57,58^. In our study, one DMR related to *syntaxin 1 A*, three DMRs related to *Snap-25* with two of them lying in promoter region, and two DMRs related to *For* were identified, and all of them were hypomethylated, implying that the transcription of these three genes might be activated through hypomethylation and act as positive regulators in learning and memory process.

*Tomosyn* and *GluCl* both play an inhibitory role in synaptic transmission. *Tomosyn* is involved in inhibiting vesicle priming and synaptic transmission^59^, and plays a role in odor memory and spatial learning and memory^60,61^. *GluCl* gene was reported to be involved in inhibitory transmission in olfactory learning and memory in the honeybees^62^. Moreover, through alternative splicing it produces two subunits to differentially regulate the olfactory memory processes of the honeybee^63^. We found that two DMRs related to *Tomosyn* and one DMR located in the promoter region of *GluCl* were hypomethylated, which might promote transcription of these two genes.

### Key genes involved in epigenetic modification

Other epigenetic mechanisms, such as histone methylation, RNA editing and miRNA, also have an effect on learning and memory process. We found that the euchromatin histone methyltransferase 1 (EHMT1, XM_003249151.1), adenosine deaminases (ADARs, XM_394309.4) and Dicer1 (NM_001123013.1), which are key enzymes for histone methylation, RNA editing and miRNA processing, respectively, were highly methylated after training.

EHMT1 is a highly conserved protein which catalyzes mono- and dimethylation of histone 3 at lysine 9 (H3K9me2) in euchromatic DNA by interacting with EHMT2^64^. It plays an important role in human cognition^65^ and mice memory^66^. The differential methylation of *Ehmt1* after learning and memory suggests that DNA methylation can regulate learning and memory by modulating the histone methylation process. Adenosine deaminases are double-strand RNA-binding proteins involved in adenosine to inosine (A to I) RNA editing. Studies in *Caenorhabditis elegans*, *Drosophila* and mouse indicated that ADAR mediated RNA editing is crucial for cognitive and behavioral correlates of nervous system function^67^. The promoter region of honeybee *Adar* gene was highly methylated after learning and memory, which might repress its transcription and reduce A- I RNA editing. Dicer1 is a type III RNAse responsible for the processing of microRNA precursors into mature miRNAs as well as long dsRNA substrates into small interfering RNAs (siRNAs)^68–70^. Mutation of *Dicer1* can enhance memory strength of mice^71^, suggesting that Dicer1 acts as an inhibitor in learning and memory process of mice. The hypermethylation of *Dicer1* after learning and memory in our study suggests that down-regulated expression of *Dicer1* promotes the learning and memory in honeybee. In a word, the DNA methylation changes of these critical genes of different epigenetic regulation implying that DNA methylation might regulate the learning and memory process through regulating other epigenetic modification process.

## Materials and Methods

### Experimental bees

The *A. mellifera* colonies used in this experiment were kept in Honeybee Research Institute, Jiangxi Agricultural University, China. The experimental bees were from a colony headed by a queen artificially inseminated with semen from a single drone to ensure a highly similar genetic background. Frames containing near emerging pupae were enclosed inside nylon-net bags and kept inside the colony overnight. The next morning the newly emerged worker bees (<12 h after emergence) were gathered into rectangular boxes and then maintained in an incubator at a constant temperature of 34 °C in a light-dark cycle of 12 h light and 12 h dark. The bees were fed daily with a mixture of 1 M sucrose and bee-bread for one week before the experiment.

### PER experiment

The proboscis extension reflex (PER) experiment began in the morning of the 8 day old bees (the day of emergence = 1 day old). The experiment procedures followed that of Letzkus^72^ and one of our previous studies^25^. Briefly, the bees were trained by a positive stimulus and a negative stimulus. The lemon odor plus 1 M sucrose solution were used as positive stimulus (reward) and vanilla odor plus saturated saline were used as negative stimulus (punishment). Twenty-four hours after training, retention tests were carried out. At this time, the bees were first given the negative stimulus, then, the positive stimulus. The bees were given three trails, those bees giving a correct response to the positive stimulus in at least two of the three trials (a very few bees didn’t extend their proboscis actively at the third trial because they ate a little more sucrose solution during the first two trials) and not giving a response to the negative stimulus in all the three trials were considered as having learned the two stimuli and good memory performers. 30 min after completion of the tests, the heads of the trained bees that have a good performance in tests and those of untrained bees (control group) were sampled and stored in liquid nitrogen. In order to get enough honeybee brain samples, six biological replicates of PER experiment were performed. For each biological replicate, the same number of learned bees and untrained bees were sampled (Table S1).

### Brain dissection

The brains were dissected from the sampled heads of honeybees under a stereo microscope using a sharp razor blade in normal saline (137 mmol/L NaCl, 2.7 mmol/L KCl, 10 mmol/L Na_2_HPO_4_, 2 mmol/L KH_2_PO_4_). The dissected brains were collected into a 1.5 ml RNase- and DNase-free Eppendorf tube placed on dry ice. Then, the sampled brain tissues were stored at −80 °C until use.

### Genomic DNA extraction

The genomic DNA was isolated from the dissected honeybee brain samples that contain brain tissues from about 50 worker bees using DNeasy Blood & Tissue Kit (Qiagen, Germany) according to its protocol. Genomic DNA degradation and contamination were checked by agarose gel electrophoresis analysis. DNA purity (ratio of OD260/280) was detected using the NanoPhotometer® spectrophotometer (IMPLEN, CA, USA) and DNA concentration was measured using Qubit® DNA Assay Kit in Qubit® 2.0 Flurometer (Life Technologies, CA, USA).

### Library preparation and quantification

Sequencing libraries were constructed for the trained and untrained groups using the above isolated genomic DNAs. For each library, a total amount of 5.2 µg genomic DNA added with 26 ng lambda DNA as negative control were randomly fragmented to 200–300 bp by sonication with Covaris S220 (Covaris, MA, USA). After purification, the DNA fragments were end repaired and were added an adenine to its two 3′ terminals. Then, cytosine-methylated sequencing adaptors were ligated to the two terminals of the DNA fragments according to the manufacturer’s instructions. After that, these DNA fragments were treated twice with bisulfite using EZ DNA Methylation-Gold^TM^ Kit (Zymo Research), followed by PCR amplification. Library concentration was quantified by Qubit® 2.0 Flurometer (Life Technologies, CA, USA) and quantitative PCR, and the length of the inserted fragments was detect using an Agilent Bioanalyzer 2100 system. Finally, the libraries were subjected to125 bp paired-end sequencing using the Illumina HiSeq. 2500 platform.

The original image data were transformed into raw sequences (Sequenced Reads) by Base Calling, and stored in FASTQ files. The distribution of sequencing error rate and bases content along reads were analyzed through in-house Perl scripts. The raw reads in FastQ format were processed using the Trimmomatic software^73^ as follows: (1) removing adaptors; (2) removing reads containing N (unknown base) more than 10% and; (3) removing the low quality reads (the percentage of the low quality bases (Phred score ≤20) ≥50%). Meanwhile, Q20, Q30 and GC content of the data were calculated using in-house scripts. The clean reads were submitted to the NCBI Sequence Read Archive database with accession number SRR5295651 for trained group and SRR5312519 for untrained group.

### Reads mapping to the reference genome

After removing the low quality reads, all the bisulfite-treated reads were aligned to the *A. mellifera* genome sequences v4.5 downloaded from NCBI (ftp://ftp.ncbi.nih.gov/genomes/Apis_mellifera) using Bismark software version 0.12.5^74^ with the default parameters. Before mapping, both the *A. mellifera* genome sequences and the clean reads were transformed into bisulfite-converted version (C-to-T and G-to-A converted) and the converted genome sequences were indexed using Bowtie2 software^75^.

Those clean reads that produce a unique best alignment from the two alignment processes (original top and bottom strand) were compared to the normal honeybee genome sequence. Then, methylation state of all cytosine positions in the reads were inferred according to the alignment results. Those identical reads that aligned to the same regions of the honeybee genome were regarded as duplicated ones. The sequencing depth and coverage of each sample were summarized using deduplicated reads. The results of methylation extractor were transformed into bigWig format for visualization using IGV browser. Sodium bisulfite non-conversion rate was calculated as the percentage of cytosines sequenced at cytosine reference positions in the lambda genome.

### Estimating methylation level

To identify the methylation site, we modeled the sum of methylated counts as a binomial (Bin) random variable with methylation rate.

A sliding-window approach was used to calculate the number of methylated and unmethylated reads with window size 3,000 bp and step size 600 bp^76^. Methylation level (ML) for each identified C site was calculated by the following formula:

$${\rm{ML}}({\rm{C}})=\frac{{reads}\,({mC})}{{reads}\,({mC})+{reads}\,({umC})}$$

Here reads (mC) represents number of reads containing a methylated C in this C site. Reads (umC) represents number of reads containing an unmethylated C in this C site. The ML value was further corrected with the bisulfite non-conversion rate according to previous studies^77^.

### Differentially methylated regions analysis

Differentially methylated regions (DMRs) were identified based on the methylation imformation of each site using the swDMR software developed by Wang *et al*.^78^ and the corresponding procedures. At first, a sliding-window approach with window size of 1000 bp and step length of 100 bp were adopted to scan the genome of the trained and untrained groups. Only windows containing more than 10 cytosine sites were retained for calculating the mean methylation level. Those windows that have fold change and difference of mean methylation level higher than 2 and 0.1 between the two samples and P value < 0.05 in the Fisher’s Exact Test were considered as potential DMRs. Repeating the above operations until all the potential DMRs genome-wide were identified, and their P values were corrected using FDR method (corrected p-value < 0.05). After that, the overlapping potential DMRs were subjected to merging and statistical test again and again, the final merged regions were regarded as candidate DMRs.

DMRs were annotated using the gene annotation file of *A. mellifera* reference genome v4.5 downloaded from NCBI (ftp://ftp.ncbi.nih.gov/genomes/Apis_mellifera/GFF) by comparing the chromosome position information of DMRs with the corresponding annotation information in the gene annotation file. When a DMR overlapped with a gene or a functional component of a gene, including promoter, 5′UTR, exon, intron and 3′UTR, it was assigned to this gene and its components. The position information of the 5′UTR, exon, intron and 3′UTR of each gene can be obtained from the gene annotation file. The promoter region contains 2 kb region upstream the transcription start site (TSS).

### GO and KEGG enrichment analysis of DMGs

Gene Ontology (GO) enrichment analysis of DMGs was performed using the GOseq R package^79^. GO terms with corrected p-value < 0.05 were considered significantly enriched by DMGs.

The KEGG pathway enrichment analysis identified significantly enriched metabolic pathways or signal transduction pathways in the DMGs by comparing with the whole-genome background.

### Data availability

The RNA-Seq data of trained group and untrained group can be obtained from the following links.

Trained group:

ftp://ftp-trace.ncbi.nih.gov/sra/sra-instant/reads/ByRun/sra/SRR/SRR529/SRR5295651/:

Untrained group:

ftp://ftp-trace.ncbi.nih.gov/sra/sra-instant/reads/ByRun/sra/SRR/SRR531/SRR5312519/).

## Electronic supplementary material

Supplementary files

Table S2

Table S3

Table S4

Table S5
