# Supplementary material for: Genome-wide DNA methylation changes associated with olfactory learning and memory in Apis mellifera
Source: Sci Rep. 2017 Dec 5;7:17017. doi: 10.1038/s41598-017-17046-1 (PMC5717273; doi:10.1038/s41598-017-17046-1)
Supplement: Supplementary file 1 — Supplementary files [file 41598_2017_17046_MOESM1_ESM.pdf]

Genome-wide DNA methylation changes associated with olfactory learning and memory in *Apis mellifera*

You Li[1], Li-Zhen Zhang[1], Yao Yi[1], Wan-Wan Hu[1], Ya-Hui Guo[1], Zhi-Jiang Zeng[1], Zachary-Y. Huang[2], Zi-Long Wang[1†]

[1] Honeybee research institute, Jiangxi Agricultural University

[2] Department of Entomology, Michigan State University

[†]Corresponding author: Zi-Long Wang, Honeybee Research Institute, Jiangxi Agricultural University, Nanchang, Jiangxi 330045, China. email: wzlcqbb@126.com

Supplementary files

Table S1 PER training results

Replicates	PER trained group		Control group
	Trained	Learned	
1	102	25	25
2	94	22	22
3	98	23	23
4	89	19	19
5	106	15	15
6	82	18	18
Total	571	122	122

Table S2 Differentially methylated regions (DMRs) between trained and untrained groups.

Table S3 DMGs between the trained and untrained groups

Table S4 GO enrichment analysis of the 963 DMGs

Table S5　Overlapped genes between the DMGs and DEGs
